# Quality of work life among primary health care nurses in the Jazan region, Saudi Arabia: a cross-sectional study

**DOI:** 10.1186/1478-4491-10-30

**Published:** 2012-09-13

**Authors:** Mohammed J Almalki, Gerry FitzGerald, Michele Clark

**Affiliations:** 1 Faculty of Public Health and Tropical Medicine, Jazan University, Jazan, Saudi Arabia; 2 School of Public Health and Institute of Health and Biomedical Innovation (IHBI), Queensland University of Technology, Victoria Park Road, Kelvin Grove, QLD, 4059, Australia

**Keywords:** Nurse, Nursing workforce, Primary health care, Quality of work life (QWL), Saudi Arabia

## Abstract

**Background:**

Quality of work life (QWL) is defined as the extent to which an employee is satisfied with personal and working needs through participating in the workplace while achieving the goals of the organization. QWL has been found to influence the commitment and productivity of employees in health care organizations, as well as in other industries. However, reliable information on the QWL of primary health care (PHC) nurses is limited. The purpose of this study was to assess the QWL among PHC nurses in the Jazan region, Saudi Arabia.

**Methods:**

A descriptive research design, namely a cross-sectional survey, was used in this study. Data were collected using Brooks’ survey of quality of nursing work life and demographic questions. A convenience sample was recruited from 134 PHC centres in Jazan, Saudi Arabia. The Jazan region is located in the southern part of Saudi Arabia. A response rate of 91% (*n* = 532/585) was achieved (effective response rate = 87%, *n* = 508). Data analysis consisted of descriptive statistics, *t*-test and one way-analysis of variance. Total scores and subscores for QWL items and item summary statistics were computed and reported using SPSS version 17 for Windows.

**Results:**

Findings suggested that the respondents were dissatisfied with their work life. The major influencing factors were unsuitable working hours, lack of facilities for nurses, inability to balance work with family needs, inadequacy of vacations time for nurses and their families, poor staffing, management and supervision practices, lack of professional development opportunities, and an inappropriate working environment in terms of the level of security, patient care supplies and equipment, and recreation facilities (break-area). Other essential factors include the community’s view of nursing and an inadequate salary. More positively, the majority of nurses were satisfied with their co-workers, satisfied to be nurses and had a sense of belonging in their workplaces. Significant differences were found according to gender, age, marital status, dependent children, dependent adults, nationality, nursing tenure, organizational tenure, positional tenure, and payment per month. No significant differences were found according to education level of PHC nurses and location of PHC.

**Conclusions:**

These findings can be used by PHC managers and policy makers for developing and appropriately implementing successful plans to improve the QWL. This will help to enhance the home and work environments, improve individual and organization performance and increase the commitment of nurses.

## Background

Quality of work life (QWL) is a complex entity influenced by, and interacting with, many aspects of work and personal life [1]. Brooks [2] argued that QWL has two goals: improving the quality of the work experience of employees and simultaneously improving the overall productivity of the organization. From a nursing perspective, Brooks defined the QWL as “the degree to which registered nurses are able to satisfy important personal needs through their experiences in their work organization while achieving the organization’s goals”. Therefore, the concept of employee satisfaction is about more than simply providing people with a job and a salary. It is about providing people with a place where they feel accepted, wanted and appreciated [3].

What is the importance of QWL? It has been argued that QWL influences the performance and commitment of employees in various industries, including health care organizations [1,4,5]. A high QWL is essential to attract new employees and retain a workforce [3]. Consequently, health organizations are seeking ways to address issues of recruitment and retention by achieving a high QWL [6]. Focusing on improving QWL to increase the happiness and satisfaction of employees can result in many advantages for the employee, organization and consumers. These include strengthening organizational commitment, improving quality of care and increasing the productivity of both the individual and the organization. According to Sirgy and colleagues [7], a happy employee is productive, dedicated and committed. On the other hand, failure to manage these factors can have a major impact on employee behavioural responses (for example, organizational identification, job satisfaction, job performance, turnover intention, organizational turnover and personal alienation) as well as outcomes of the organization [7].

Reviewing previous studies of QWL identified differing numbers of factors that have an impact on the QWL of nurses. One such factor was the lack of work-life balance [8-10]. In a number of recent research studies among nurses in the USA [8,9], Iran [10,11] and Taiwan [1], rotating schedules were found to negatively affect their lives so they were unable to balance work with family needs. Additionally, nurses thought on-site child care and day care for the elderly were important for their QWL [8-10]. The nature of nursing work was another factor that affects the QWL of nurses. The results of existing studies on the QWL of nurses indicated dissatisfaction of nurses in terms of heavy workload, poor staffing, lack of autonomy to make patient care decisions, and performing non-nursing tasks [8-10].

Another factor that influences the QWL of nurses is the work context, including management practices, relationship with co-workers, professional development opportunities and the work environment [8-10,12]. Potential sources of dissatisfaction with management practices include lack of participation in decisions made by the nurse manager, lack of recognition for their accomplishments, and lack of respect by the upper management [8,10]. Reported findings regarding co-workers and the QWL of nurses are inconsistent. While some studies found nurses to be satisfied with their co-workers including physicians [8], others reported the opposite. A study of nurses in Saudi Arabia found they were dissatisfied with the relationship with their co-workers, especially physicians [13], where they experienced low levels of respect, appreciation and support. Additionally, they had poor communication and interaction with physicians. Prior research also indicated the impact of professional development opportunities such as the promotion system, access to degree programs and continuing education on the QWL of nurses [1,10,14]. In terms of work environment, results from a wide variety of studies found that nurses were dissatisfied with the security department with resultant concerns about safety in the workplace [8,10,13,15]. Additionally, inadequacy of patient care supplies and equipment is related to dissatisfaction of nurses and other health professionals. A number of health care studies in Saudi Arabia indicated insufficiency of patient supplies, especially in primary health care (PHC) facilities [16-19].

External factors such as salary and the image of nursing were of concern in the literature regarding the QWL of nurses [1,10], and were reported sources of dissatisfaction for nurses in various organizations and countries [20-25]. Lewis and colleagues [26] found that extrinsic predictors of QWL such as pay and financial benefits explained 40% of the variance in QWL satisfaction.

An examination of prior literature of the QWL of nurses highlighted a dearth of QWL studies among PHC nurses, despite their important role in providing preventive and curative health care. In Saudi Arabia, the PHC centre is recognized as the basic health unit or the first point of contact between the community and the health care system. It provides a set of health care services including prevention programs, treatment of simple diseases, chronic care management, maternal and child health services (including immunization), environmental health, and public health education [27]. PHC services provide a large part of the basic health care to the Saudi community. According to the Ministry of Health [28], 82% of the total visits to the Ministry of Health institutions occurred in PHC centres. Therefore, studies that assess the perception of QWL among PHC nurses are very important. This importance increases in the light of the increased development of PHC services, which is accompanied by nurse shortages [29]. This study is part of a larger study aiming to improve the retention of PHC nurses in the Jazan region, Saudi Arabia through exploring and assessing their QWL and turnover intention. The purpose of the present paper, therefore; is to report about the QWL among PHC nurses in the Jazan region, Saudi Arabia. Other parts of the whole research will be reported elsewhere.

## Methods

### Design and sample

A descriptive research design, namely a cross-sectional survey, was used in this study. The research was performed at the PHC centres in the Jazan region, Saudi Arabia, between April and July 2009. The Jazan region is located in the southern part of Saudi Arabia. The total number of targeted PHC centres in Jazan was 134 [30]. They are distributed throughout urban and rural areas and, at the time, employed about 585 Saudi and non-Saudi nurses. All registered nurses working in these centres were eligible to participate in this study.

### Instrument

In addition to the demographic information, the research instrument used in this study was the Brooks’ survey of quality of nursing work life. This questionnaire was developed by Brooks [2] to measure the QWL among registered nurses. It is a self-completion questionnaire with 42 items divided into four subscales: (a) work life/home life, (b) work design, (c) work context and (d) work world. The work life/home life dimension is defined as the interface between the work and home life of the nurse. The work design dimension is the composition of nursing work and describes the actual work that nurses perform. The work context dimension includes the practice settings in which nurses work and explores the impact of the work environment on both nurse and patient systems. Finally, the work world dimension is defined as the effects of broad societal influences and changes on the practice of nursing [2,31]. The instrument asks respondent nurses how much they agree or disagree with each item on a 6-point scale, with 1 indicating ‘strongly disagree’, 2 ‘moderately disagree’, 3 ‘disagree’, 4 ‘agree’, 5 ‘moderately agree’, and 6 ‘strongly agree’. The test-retest reliability was determined in a traditional 14-day manner with Pearson’s *r* = 0.90 (*n* = 53), where 1.00 indicated ‘perfect’ reliability. The construct validity was calculated for the 42 item survey using Cronbach's alpha, r = 0.89 (n = 265), where 1 indicates 'perfect' validity [2]. In this study, the Cronbach’s alpha for Brooks’ scale is 0.89.

The Brooks’ survey of quality of nursing work life has been used by other published works in the USA and Iran [9,10,12,32], with increasing global interest. According to Clarke and Brooks [33], requests to use Brooks’ survey have been received from researchers and graduate students in Greece, Estonia, Canada (Ontario, Quebec), India, Iran, Australia, Malaysia, Turkey, and Taiwan.

To ensure the suitability of the used tools to the purpose of the present study, the questionnaire was contextualized to meet the local context and the multicultural environment of the PHC nursing workforce in the Jazan region. In addition to the original English format, the questionnaire was translated into Arabic using a translating and back-translating technique and a committee approach [34,35]. A bilingual researcher blindly translated the instrument from English to Arabic and a second bilingual researcher back-translated it independently [35]. A panel of three bilingual experts in health care workforce management and in health research reviewed the translated questionnaire in compared to the English format and assured its validity [36]. Two pilot studies were conducted to ensure the appropriateness, structure and clarity of the questions. The first pilot study involved seven registered nurses from one PHC centre. Following recommendations from participants, a few minor changes to the demographic questions were performed. The revised questionnaire was administered to a sample of 59 PHC nurses. No comments regarding the difficulty of terminology, question structure or item clarity were reported by the participants of the second sample. Demographic data were collected for gender, age, marital status, dependent children, dependent adults, nationality, education level, nursing tenure, organizational tenure, positional tenure, payment per month and location of PHC.

### Data collection and analysis

Following ethics approval from Queensland University of Technology and permission from the Ministry of Health in Saudi Arabia, the survey was sent to PHC nurses through the Department of Primary Health Care in Jazan. A coded return envelope and a cover letter were also sent that further explained the research, provided contact details of the researchers and illustrated the steps taken to maintain confidentiality. Participants were advised about the voluntary nature of participation. The return of the completed questionnaire was accepted as an indication of the participant’s consent to participate in this study. Every 2 weeks, the PHC managers were reminded about the study by phone or letter. The participants were advised of the protective procedures in place to ensure anonymity. No names or other identifiable information were needed on the questionnaires which were sealed by participants and placed in individual envelopes (provided) upon completion. Data were analysed using SPSS version 17 for Windows (SPSS, Inc., Chicago, IL). Descriptive statistics, total scores and subscores for QWL items and item summary statistics were computed and reported. Other tests include *t*-test and one way-analysis of variance (ANOVA).

## Results

### Sample profile

The questionnaire was distributed to 585 PHC nurses. The overall response rate was 91% (n = 532); however, the effective response rate after removing incomplete questionnaires was 87% (n = 508). This high response rate suggests that response bias will be minimal. The majority of respondents were females (*n* = 342, 67.3%), Saudi (*n* = 367, 72.2%), Arab (*n* = 375, 73.8%), aged between 20 and 29 years (*n* = 224, 44.1%), married (*n* = 375, 73.8%), with children (*n* = 310, 61%) and dependent adults (*n* = 279, 54.9%). Most of the respondents held less than a Bachelor Degree in nursing (n = 477, 93.9%) and received a monthly salary of 5,000 to 10,000 Saudi Riyals (SR) (1US$ = SR 3.75) (*n* = 235, 46.3%). About 52% of respondents were working in rural areas (n = 265), as staff nurses (*n* = 471, 92.7%), during morning working period (*n* = 440, 86.6%), for 45 hours per week (*n* = 331, 65.2%) and covered caseloads which included male clinics, female clinics, maternity and childhood, immunizations, home visits, chronic diseases, medical records and management. About two thirds (62%) of the respondents stated that they cover two departments or more during their duties. The mean work experience as a registered nurse was 11.3 years, with about 6.6 years in the current PHC centre and 6.1 years in the current position.

### Demographic variables and quality of work life

An independent samples *t*-test and an ANOVA were conducted to determine any significant difference in the QWL scores by demographic variables. Significant differences were found according to gender, age, marital status, dependent children, dependent adults, nationality, nursing tenure, organizational tenure, positional tenure, and payment per month. The eta squared test for these demographics indicates small to medium effect size of the variation in QWL scores. No significant differences were found according to education level and location of PHC. Results of *t*-test and ANOVA procedures are presented in Table 1.

**Table 1:** **Quality of work life by demographic variables using *t*-test and analysis of variance**

**Variable**	**Mean**	**SD**	**t/F value**	***P*value**
**Gender**				
Male	134.65	25.62	−3.11	0.002
Female	141.81	20.77		
**Age**				
20-29 years	134.35	24.46	10.46	0.000
30-39 years	139.46	21.62		
40-49 years	147.42	16.65		
50-59 years	151.85	16.44		
**Marital status**				
Never married	133.26	26.26	6.49	0.002
Married	141.25	20.84		
Divorced/widowed	147.75	30.24		
**Dependent children**				
Yes	141.97	21.03	3.11	0.002
No	135.55	24.65		
**Dependent adults**				
Yes	136.87	25.20	−2.87	0.004
No	142.67	18.94		
**Nationality**				
Saudi	137.76	23.51	−2.69	0.007
Non-Saudi	143.83	19.93		
**Education level**				
Institute	139.72	24.76	1.85	0.138
Diploma	138.69	21.65		
Associate	138.06	21.50		
Bachelor or higher	149.42	21.07		
**Nursing tenure**				
≤ 4 years	131.15	25.64	16.21	0.000
5-9 years	138.95	22.71		
≥ 10 years	144.54	19.33		
**Organizational tenure**				
≤ 4 years	135.17	24.88	11.62	0.000
5-9 years	143.24	18.95		
≥ 10 years	145.66	17.97		
**Positional tenure**				
≤ 4 years	135.44	24.67	11.13	0.000
5-9 years	143.61	19.20		
≥ 10 years	145.83	18.06		
**Payment per month**				
< SR 5,000	141.74	20.68	5.05	0.007
SR 5,000-10,000	135.83	25.49		
> SR 10,000	143.13	18.83		
**Location of PHC**				
Urban	139.94	21.00	0.46	0.647
Rural	139.01	24.18		
SR, Saudi Riyal.				

### Rating of quality of work life among primary health care nurses

The total possible score for the Brooks’ scale can range from 42 to 252. A low total scale score indicates a low overall QWL, while a high total score indicates a high QWL. The actual range score of the current study was 45 to 218 (mean = 139.45), which is lower than the average score on the Brooks’ scale. This finding indicated that the respondents were dissatisfied with their work life [8]. The mean of the work life/home life and the work context subscales were lower than average: 18.97 and 66.25, respectively. For the work design and work world subscales, means of the actual range were almost equal to the average score, suggesting that respondents were not highly pleased with each dimension. Table 2 shows the possible range scores, average, actual range scores and means for total scale and subscales. 

**Table 2:** Total scores and subscores for quality of work life items

**Scale**	**Possible range**	**Average**	**Actual range**	**Mean**	**SD**
42-Item scale	42–252	147	45–218	139.45	22.7
7-Item work life/home life subscale	7–42	24.5	8–37	18.97	5.15
10-Item work design subscale	10–60	35	11–54	35.66	6.72
20-Item work context subscale	20–120	70	20–105	66.25	12.4
5-Item work world subscale	5–30	17.5	5–29	18.69	3.6

### Describing quality of work life among primary health care nurses

Following the strategy used by Brooks and Anderson [8], the 6-point scale was collapsed to two categories: agree and disagree. The agree category contains positive responses (agree, moderately agree and strongly agree), while the disagree category contains negative responses (strongly disagree, moderately disagree and disagree). Table 3 shows the number and percentage of nurse respondents for each category. 

**Table 3:** Factors influencing the quality of work life among primary health care nurses

	**Items**	**Agree**		**Disagree**	
		**No.**	**%**	**No.**	**%**
**A**	**Work life/home life dimension**				
	Energy left after work	150	29.5	358	70.5
	Policy for vacations is appropriate for me and for my family	211	41.5	297	58.5
	Ability to balance work with family needs	213	41.9	295	58.1
	Important to have support for taking care of elderly parents*	242	47.6	266	52.4
	Important to have on-site/near child care services*	401	78.9	106	20.9
	The system of working hours negatively affects my life*	409	80.5	99	19.5
	Important to have on-site ill child care services*	420	82.7	88	17.3
**B**	**Work design dimension**				
	Enough registered nurses	111	21.9	397	78.1
	Quality assistance from nursing assistants and service workers	144	28.3	364	71.7
	Many interruptions during daily work routine*	173	34.1	334	65.7
	Many non-nursing tasks*	197	38.8	311	61.2
	Sufficient assistance from nursing assistants and service workers	198	39.0	310	61.0
	Workload is too heavy*	203	40.0	305	60.0
	Autonomy to make client/patient care decisions	313	61.6	193	38.0
	Ability to provide quality client/patient care	376	74.0	132	26.0
	Enough time to do jobs	424	83.5	84	16.5
	Satisfaction with job as a PHC nurse	454	89.4	54	10.6
**C**	**Work context dimension**				
	**Management and supervision**				
	Recognition of accomplishments	161	31.7	347	68.3
	Nurse manager/supervisor provides adequate supervision	221	43.5	287	65.5
	Participate in decisions made by nurse manager/supervisor	188	37.0	320	63.0
	Upper-level management has respect for nursing	196	38.6	312	61.4
	Enough feedback by nurse manager/supervisor	198	39.0	310	61.0
	Nursing policies and procedures facilitate the work	248	48.8	260	51.2
	Good communication with nurse manager/supervisor	328	64.6	180	35.4
	**Co-workers**				
	Availability of teamwork	335	65.9	173	34.1
	Good communication with physicians	433	85.2	75	14.8
	Respect by physicians	435	85.6	72	14.2
	Good communication with other co-workers	443	87.2	65	12.8
	Friendships with co-workers	453	89.2	55	10.8
	**Development opportunities**				
	Support to attend continuing education/training programs	140	27.6	368	72.4
	Career advancement opportunities	147	28.9	361	71.1
	Important to have the opportunity to further nursing education*	473	93.1	35	6.9
	**Work environment**				
	Security department provides secure environment	202	39.8	306	60.2
	Adequate client/patient care supplies and equipment	203	40.0	305	60.0
	Safe from personal harm at work	280	55.1	228	44.9
	Belong to the workplace	371	73.0	137	27.0
	Important to have break area for nurses*	446	87.8	62	12.2
**D**	**Work world dimension**				
	Society has an accurate image of nurses	124	24.4	384	75.6
	Ability to find same job in another organization	193	38.0	315	62.0
	Salary is adequate	196	38.6	312	61.4
	Job is secure	389	76.6	119	23.4
	Nursing work positively impacts lives of others	482	94.9	26	5.1

* Reversed items. PHC, primary health care.

### Work life/home life dimension

The majority of nurse respondents indicated they were not satisfied with items in the dimension of work life/home life. Approximately 83% (*n* = 420) of respondents reported their need to have on-site child care services for sick children during working hours, and 78.9% (*n* = 401) agreed that it is important to have on-site/near childcare services. More than 80% (*n* = 409) agreed that they are not happy with working hours which do not suit their daily life, 70.5% (*n* = 358) stated that they have no energy left after work and over half (58%, *n* = 295) of respondents were not able to balance work with their family needs. Over half (58.5%, *n* = 297) of respondents felt that the policy of their PHC centres for vacations was inappropriate neither for nurses nor for their families.

### Work design dimension

Factors relating to the nursing workforce were the most influential factors in the work design dimension. Seventy-eight percent (*n* = 397) of respondents indicated that there are not enough registered nurses in their PHC centres and only 28.3% (*n* = 144) of nurse respondents agreed that they have quality assistance from the supporting personnel (nursing assistants and service workers). Forty percent of respondents (*n* = 203) found that their workload is heavy. More than one third (38%, *n* = 193) of respondents reported that they do not have autonomy to make client/patient care decisions. Even in light of these results, 89.4% (*n* = 454) feel satisfied as PHC nurses.

### Work context dimension

Management and supervision issues were of concern. Approximately 65% (*n* = 287) of respondents reported that they do not receive adequate supervision from their nurse manager/supervisor, 61% (*n* = 310) do not receive enough feedback regarding their performance and only 31.7% (*n* = 161) felt recognized for their accomplishments. Sixty-three percent (*n* = 320) stated that they have no chance to participate in decision-making processes. Additionally, more than half of the nurse respondents (51.2%; *n* = 260) stated that the nursing policies and procedures are not supportive enough, and only 38.6% (*n* = 196 ) of the nurses in this sample felt respected by the upper-level management; however, 64.6% (*n* = 328) perceived that they have good communication with the management department.

In terms of professional development opportunities, 93.1% of the respondent nurses (*n* = 473) agreed that it is important to have the opportunity to further their nursing education without leaving the current job, 72.4% (*n* = 368) claimed that they do not receive support to attend continuing education and training programs and 71.1% (*n* = 361) reported that their work organizations do not provide adequate opportunities for career advancement.

More positively, nurses were notably satisfied with factors relating to their co-workers. Eighty-nine percent (*n* = 453) stated that they have good friendships and relationships with their co-workers. The majority of respondents (87.2%, *n* = 443) and (85.2%, *n* = 433) agreed they have good communication with other co-workers and physicians, respectively, with only 14.2% (*n* = 72) of respondents feeling not respected by physicians. Approximately 65.9% (*n* = 335) reported that there is teamwork in their organizations.

Regarding the working environment, approximately 60% (*n* = 306) felt that the security department did not provide a secure working environment, and about 45% (*n* = 228) felt unsafe in relation to personal harm (physical, emotional, or verbal) at work. Unexpectedly, only 40% (*n* = 203) reported that they have adequate client/patient care supplies and equipment. Nurses also reported the importance of having a private break area (87%; *n* = 446) where they could have some time away from patients.

Despite expressing that they were not satisfied with many working factors, the majority of respondents (73%; *n* = 371) expressed a sense of belonging in their workplace. 

### Work world dimension

About three-quarters (75.6%, *n* = 384) of the nurses in this study did not think society has an accurate image of nurses. Ninety-five percent (*n* = 482) of nurses, however, believed that nursing work positively affects the lives of others, indicating excellent attitudes towards their career as well as an exceptional sense of self-image. Payment was also an essential factor that contributes to dissatisfaction among PHC nurses. Approximately 61% (*n* = 312) stated that their salary is not adequate given the job market condition and the nature of roles they are performing. Although members of the nursing profession in Saudi Arabia are in critical demand, 62% (*n* = 315) of respondents think that they will not be able to find a similar job in another organization easily; however, almost 77% (*n* = 389) believe that their jobs are secure so they do not expect to lose them unexpectedly.

## Discussion

The purpose of this study was to assess the QWL among PHC nurses in the Jazan region, Saudi Arabia. The findings of this study indicated a number of factors of concern regarding the QWL among PHC nurses.

### Perception of the quality of work life among primary health care nurses

The PHC nurses were asked to rate their QWL. The aim was to gain an understanding of the QWL of PHC nurses by assessing their work life experience. Contrary to the Brooks and Anderson study [8], where respondents were pleased overall with their work life situations, the findings of the present study indicated that the respondents were dissatisfied with their work life. However, these findings are consistent with findings of a number of previous studies where nurses were not satisfied with their work life [9-11,20]. Efficient QWL programs can improve the morale of employees and organizational effectiveness [37]. QWL can also improve the quality of nursing care and retention of nurses [33,38]. Improving QWL may be a more practical and long-term approach to attracting and retaining the workforce that should be considered by health care managers [4].

The majority of nurses in this study perceived dissatisfaction with the work life/home life factors including family needs, working hours and energy left after work. Nurses reported that they spent a long time at work so they had little energy left after work. As a result, the nurses were unable to balance their work with their family life. This is consistent with findings from previous studies [8,10]. According to Ashy [39] “many working Saudi women have to work hard to balance the demands of their careers and their families.” The standard working hours for PHC professionals, including nurses, are 47.5 hours per week raised to 49 hours in PHC centres that work two periods per day, compared to 35 hours for other workers in all other public sector occupations [40]. However, nurses only receive 20% extra in their salary, compared to 45% for pharmacists and 70% for physicians, in recognition of these additional hours [41]. When nurses found the demands of work incompatible with a fulfilling home life, their turnover intention become more pronounced [42,43]. Lack of support for the family members of nurses (that is, children and adult dependents) and inadequacy of vacations were other sources of unsatisfactory nursing work life. These findings are in-line with previous literature [8-10].

In keeping with global trends, a shortage in the nursing workforce in PHC was identified as a main problem in the current study. This shortage puts a high load on the nurses who remain in PHC settings. Issues of poor staffing and demanding workloads among nurses were found to be ‘push’ factors for nurses considering leaving their organizations [44,45]. Lack of support for the family members of nurses (that is, children and adult dependents) and inadequacy of vacations were other sources of unsatisfactory nursing work life. These findings are in-line with previous literature [8-10]

Despite the shortage of PHC nurses, the nurses in this study were given additional non-nursing tasks. This malutilization of the nursing workforce may increase the shortage of nurses and affect their nursing skills and experience. Such challenges may put significant pressure on nurses, affecting their perceptions towards their work lives [46]. Approximately one third of respondents reported that they do not have the required autonomy to make client/patient care decisions. Autonomy of practice in nursing was found to be associated with quality of care and job satisfaction [47-49].

Management practices were identified as one of the problematic areas in the ‘work context’ dimension. There is a lack of supervision, feedback, participation in decision making and respect shown by upper-level management. Additionally, working policies and procedural guidelines are inadequate. Of concern, nurses were not recognized for their efforts and accomplishments. In previous studies, nursing management practices were found to be associated with the quality of care, employee productivity, employee satisfaction and the intent to stay or leave [44,50-52]. Bodek [53] argued that employees want to feel respected at work for what they do and who they are. Above all, they need to feel valued for their skills, knowledge, performance and participation in the development process. According to AbuAlRub and Al-Zaru [54], recognition of the performance of nurses has a direct effect on the level of intention to stay at work. Working hard without appreciation can intensify the turnover intention among registered nurses.

Opportunities for professional development (that is career advancement, opportunity to further nursing education, and access to continuing education) were reported by the respondents as unsatisfactory. This finding was supported by prior nursing research [22,55]. According to Cabigao [22] insufficient opportunities for professional development often diminish nurses efforts to provide quality care and is a major reason for their job dissatisfaction. Hart [56] found that nurses who were enrolled in an educational program were less likely to leave their positions than those who were not enrolled in any program. In terms of continuing education, 72% of nurse respondents stated that they did not receive support to attend continuing and in-service education programs. Similarly, Alhusaini [13] found that 30.3% of nurses in Riyadh were not offered any training courses or continuing education programs and 65.9% were offered very short courses (1 to 5 days per year). Nurses, as health care professionals, seek to continually refresh their knowledge and skills to provide quality patient and community care and to satisfy their QWL. A lack of training programs for nurses will impact on their competence and performance [13]. Dissatisfaction with career advancement was also reported in other nursing studies [8,10,57,58]. Alhusaini [13] found that the lack of role clarity is one of the noticeable work obstacles for Saudi nurses. Hence, there is a need to establish an appropriate career ladder or rank system for PHC nurses.

The working environment was also of concern among PHC nurses. More than half of the respondents reported that the security department does not provide a secure working environment. A number of previous nursing studies highlighted concerns about the safety of the working environment as a major factor in nurses’ dissatisfaction with their workplaces [8,10,13]. El-Gilany and colleagues [15] conducted a survey study in Al-Hassa, Saudi Arabia, to highlight the incidence of workplace violence against 1,091 PHC workers. About 28% were exposed to at least one violent event during the past year. Emotional and physical violence accounted for 92.1% and 7.9% of violent events, respectively. Feeling safe at work is essential for nurses to perform their work appropriately. In accordance with prior nursing research [13,57], the majority of nurses in this study were not happy with their break area (recreation room). They do not have a particular place to rest, eat or pray. Private and furnished break areas for PHC nurses are essential for their comfort in the workplace. More important to the working environment experience, nurses reported a lack of client/patient care supplies and equipment. Availability of supplies and equipment is essential for providing quality health care. A number of PHC studies in Saudi Arabia revealed that many essential resources for health care were not adequately available [16,18,19]. Lack of essential patient care supplies may impact on the level of QWL of nurses and their performance and productivity. Nurses need more efficient and effective working environments, which ensure that patients become the priority and patient needs are met [59].

Many nurses in this study felt that people do not have an accurate image of the nursing profession. In Saudi Arabia, nursing is not ranked as highly as other medical jobs, such as medicine and pharmacy [60,61]. According to Al Thagafi [61], the public does not appreciate the role of nurses in providing health care, believing that nurses are no more than the assistants to physicians. Alamri and colleagues [62], however, found that people in Saudi Arabia understand the importance of nursing and they believe jobs must be occupied by locals; however, for their young, they prefer high prestige occupations such as medicine [62,63]. This view of nursing in Saudi Arabia is in-line with other countries such as Iran, Japan, Jordan and Kuwait [10,23,24,64]. Public stereotypes are found to negatively affect nursing practice and retention [65,66].

Payment including salary and financial incentives was found to be a major factor in the dissatisfaction of nurses with their QWL (61.4%). Although several research studies found that payment is not the prime motivator for employees [67], behavioural theorists such as Herzberg [68] and Maslow [69] suggest that satisfying basic needs is essential because people cannot concentrate on their higher needs until basic needs are met [1]. In support of this, several recent nursing studies have found that salary, financial benefits and equity in pay were very important to nurses [1,20,46,70-72], and the lack of such benefits may impact on the satisfaction, commitment and performance of affected employees [11,73-75].

The majority of respondents in this study (76.6%) reported that their jobs are secure and they do not expect to lose them unexpectedly. This result appears at odds with research conducted overseas [10,74]. Additionally, the majority of respondent nurses had a high belief in the value of the nursing profession. In contrast, a study of 346 hospital nurses in Saudi Arabia found that only about one third of the sample had a high perception of nursing [76]. A high perception of the profession and the personal interest in PHC nursing as well as the sense of belonging in the workplace among respondents of the current study are noteworthy for the nursing directors, PHC managers and health care policy makers to maintain their nursing workforce through improving QWL.

### Demographic variables and quality of work life

Significant differences in the QWL were found according to gender, age, marital status, dependent children, dependent adults, nationality, nursing tenure, organizational tenure, positional tenure, and payment per month. Male nurses had significantly lower mean scores of job satisfaction than female respondents, which is similar to other studies [77,78]. As 99% of male respondents in this study were Saudi nurses, this result could be attributed to the poor image of the nursing profession in Saudi Arabia [61]. Older nurses had significantly higher mean scores of QWL than younger nurses. Likewise, nurses with more years of nursing experience and time spent at their current PHC organization and position were more satisfied with their QWL than those with less experience. Many studies have shown that older and more experienced nurses are more satisfied than younger and less experienced nurses [77,79-83]. This may be attributed to the ability of older nurses (as mature age-wise) to make a better adjustment to the work environment when compared with younger nurses [80]. The years of experience may increase the familiarity and competence of nurses as well as their understanding of the work-related expectations [77,82]. Moreover, in the Arab culture, older and experienced employees are accorded greater recognition by managers, and therefore they tend to be more satisfied [80]. When marital status was considered, never-married nurses were found to have significantly lower mean scores of QWL than other peers, a finding both consistent [84] and inconsistent [81,85,86] with previous research studies. One possible explanation for the finding may be that the never-married nurses were younger compared to other groups so they may not have the required skills to cope with challenges at work when they differ from expectations. Another explanation is that the majority of married nurses may have been living with their families, which contributed significantly to their work satisfaction [84]. Although majority of nurses were asking for childcare services by their employees, respondents with children were more satisfied with their QWL than those who had no children. Presence of children in the life of PHC nurses may increase their responsibilities and in turn encourage their stabilization and job satisfaction. Nurses with dependent adults were less satisfied with their QWL compared to those without dependent adults. A number of previous nursing studies reported on the impact of dependent adults on the satisfaction of employees regarding their QWL [8-10]. In Saudi Arabia, according to the Islamic context and the local culture, offspring have clear obligations towards their parents and relatives. However, employers in Saudi Arabia do not provide any support services for employees with dependent adults. In respect to nationality, non-Saudi nurses were found to have significantly higher mean scores of QWL when compared with Saudi nurses. However, previous research has been controversial in this regard. While a number of studies found a significant relationship between employee satisfaction and nationality [77,84,87], others revealed no association [81,86]. Why the Saudi nurses were less satisfied than their counterparties is not clear. However, this could be attributed to their general perception towards their work life, including family needs, professional development, work environment, work conditions, public image of nursing and financial benefits. An investigation into the monthly salary level as a demographic variable revealed a significant difference in scores of nurse satisfaction with the QWL. While this result is inconsistent with findings of a previous nursing study in Saudi Arabia [86], it is in-line with study findings elsewhere [88]. Finally, although many previous studies support the notion that highly educated individuals develop higher satisfaction with their work [89-91], this study did not reveal any significant differences in the QWL scores of nurses related to educational level. However, based on the mean scores, it was observed that nurses with a Bachelor Degree or postgraduate qualification had higher QWL scores.

### Recommendations

Based on the findings of the present study, key suggestions are proposed to improve QWL of PHC nurses and consequently the quality of care provided in PHC facilities.

Nursing executives and PHC managers need to consider the family aspect of their registered nurses. Childcare facilities, support for nurses who have elderly parents, convenient working hours, and sufficient vacations should be made available for nurses. These advantages will help nurses to balance work with their family requirements.

More qualified registered nurses and sufficient and trained support personnel (that is, nursing assistants and service workers) as well as an equitable distribution of the current nursing workforce are needed to reduce workload, and to ensure adequate nursing services for patients, families and the community, particularly in an era of a transition into family medicine and PHC services. The PHC department could attract unemployed graduate nurses from private health institutes, subject to appropriate preparation for the field of PHC. However, to achieve this, PHC organizations should be financially independent. Currently, the funding policy is governed by the Ministry of Health.

PHC managers and nursing leaders should consider partnerships with relevant departments and educational organizations to offer part-time and distance-learning opportunities to enable nurses to further their education and develop their nursing knowledge and skills while working in PHC centres, especially those in rural areas. Additionally, PHC organizations should run free-of-charge continuing nursing programs and various training workshops at PHC centres and assist staff to attend training provided by other organizations.

PHC managers and policy makers should encourage the professional growth of PHC nurses through the provision of a systematic career ladder. Currently, there is no significant difference in roles and positions of PHC nurses, irrespective of their qualifications or experience.

For the comfort of nurses, they should be provided with a furnished break area where they can rest and be able to place their private belongings securely. Additionally, the security of the PHC working environment must be improved such as through the introduction of security departments as in other health care organizations. Finally, to provide quality nursing care, PHC centres must be supported with the required materials and equipment for health care services. Nurses need working environments that meet the needs of patients, employees and providers.

PHC managers should work with the media to demonstrate the vital role of PHC nurses in the care of the community, in the provision of health care services and in the advancement of the health of the population.

The current salary system is problematic for PHC nurses. The salary of nurses should be increased commensurate with the tasks performed. Nurses also should be provided with fair financial benefits such as allowances for housing, working in remote areas, dealing with infectious diseases, or working in open public areas. Other health professionals in Saudi Arabia receive several of these benefits.

Most nurses in this study were not satisfied with management practices. Nurse managers/supervisors should be provided with short training programs on the art of management, leadership and communication skills. Approaches should be developed to allow nurses to participate in decision making regarding practices that influence their work life, receive meaningful feedback on their performance and recognition for their accomplishments. Finally, adequate job descriptions, working policies and standard procedures for PHC nursing practice are urgently required.

More social, managerial, professional and organizational support should be directed to young and novice nurses who were found in this study to be less satisfied than experienced nurses. Older nurses may require more sense of appreciation, valuation and respect.

### Suggestions for future research

The current study used a cross-sectional survey design which limits the observation of change over time. There is a need to conduct longitudinal research using a few selected PHC organizations to gain an in-depth understanding of the determinants of and changes in QWL of PHC nurses at various points in time. An intervention study to improve QWL of PHC nurses using the findings of the current study is required. A comparative study between PHC centres and hospitals as well as public sector and private sector organizations in terms of QWL of nursing personnel is required. Such a study may assist in identifying the determinants of QWL in each sector that may be different from sector to sector according to differences in the working system and environment. A further comparative study regarding QWL between nurses and other health professionals in PHC services is required.

### Limitations

A number of limitations in the current study have been identified. The sample was drawn from nurses who were willing to participate in the study. Although all of the PHC centres (n = 134) in the Jazan region were included in this research, the voluntary sampling methodology may limit the generalizability of the findings. However, the high response rate (effective response rate = 87%) suggests that response bias is minimal. The information was gathered through a self-reporting survey leaving the interpretation to the participant. The use of self-reporting instruments may have decreased the reliability of responses due to misinterpretation of some of the items. The questionnaires were distributed to the PHC nurses through their managers - this strategy could have allowed the managers to pressure (intentional or unintentional) registered nurses to complete the survey in a particular way [74]. However, there were no reports of pressure placed on respondents from managers. Despite these limitations, the findings of the study provide important contribution to the existing research on the QWL, particularly for PHC nurses.

## Conclusions

The purpose of this study was to assess the QWL among PHC nurses in Jazan, Saudi Arabia, and to identify major influencing factors. Findings from this study suggest that PHC nurses are not satisfied with their QWL. Additionally, the findings revealed many areas of the work life of nurses in PHC that require planned reform. These include the family needs of nurses, working hours, nursing staffing, autonomy of practice, management and supervision, professional development opportunities, working environment, attitudes of public towards nursing and salary factors. Knowing the factors influencing the work life of PHC nurses should assist the development of effective strategies to improve their QWL. What is positive in these findings is that the majority of respondents are satisfied to be nurses and they felt a sense of belonging in their workplaces. It is contended that if other factors can be addressed, PHC will attain an outstanding nursing workforce and, in turn, will ensure the quality of services provided. The results of this study provide baseline information in understanding the work life of nurses in PHC facilities, particularly in Saudi Arabia.

## Abbreviations

ANOVA: analysis of variance; PHC: primary health care; QWL: quality of work life; SR: Saudi Riyal.

## Competing interests

The authors declare that they have no competing interests.

## Authors’ contributions

All of the authors have contributed significantly to the development of this work. MJA contributed to the design, collected and synthesized the data used in this paper and wrote the main text. GF and MC contributed to the design, editing and revisions of the manuscript. All authors read and approved the final manuscript.
